# Recent Advances: Heterocycles in Drugs and Drug Discovery

**DOI:** 10.3390/ijms25179503

**Published:** 2024-08-31

**Authors:** Ivana Pibiri

**Affiliations:** Department of Biological, Chemical and Pharmaceutical Sciences and Technologies, University of Palermo, 90128 Palermo, Italy; ivana.pibiri@unipa.it

Interest and research focusing on the design of novel pharmaceutical agents is always growing. Heterocycles, a class of chemical compounds containing at least one atom other than carbon in the ring structure, have long been recognized as recurrent scaffolds in medicinal chemistry for their significance in drug discovery and development.

Their structural diversity and versatility make them attractive building blocks exploited in drug design as antibiotics, antivirals, antifungals, antitumoral, antioxidants, neuroprotective agents, and so on, paving the way for innovative therapies and improved patient outcomes.

This Special Issue aims to highlight some of the recent advances in this exciting field by collecting recent developments of the research on bioactive heterocyclic compounds. 

In the last few years, advancements in synthetic methodologies and computational tools have offered easy synthetic accessibility, allowing to rationally design and optimize heterocyclic compounds with enhanced biological activities through a deeper understanding of structure–activity relationships. This has led to the development of promising new drug candidates with improved therapeutic profiles.

Just to cite a few recent examples, among the most interesting structures, triazoles, which are bioisosters of the amide bond [1], play a pivotal role in drug–target interactions due to their polarity, potential improvement in water solubility, and intrinsic metabolic stability [2].

Benzimidazoles have been widely studied and recognized as bioactive compounds with great potential in the drug market, a reported example concerns bis–benzimidazole derivatives as new and selective inhibitors of arginase from *Leishmania mexicana* [3].

Pyrazoline derivatives, among the most interesting scaffolds recognized as bioactive compounds, are studied as antitumor compounds [4].

Besides those containing nitrogen, pharmacologically interesting heterocycles extend to oxacycles such as pyran and tetrahydrofuran, widely represented structural motifs in approved drugs [5]; in this respect, a recent review focused on pyran-based biologically active compounds as anti-tumor and anti-viral agents [6].

Medicinal chemists have exploited furan-related compounds to create numerous innovative antibacterial agents with remarkable therapeutic efficacy in order to combat the enduring issue of microbial resistance [7].

Also, in this context, the synthesis and antibacterial activity of mono- and bi-cationic pyridinium 1,2,4-oxadiazoles, and triazoles have been reported [8].

Moreover, among small molecules, oxadiazoles have been explored as readthrough promoters, able to rescue protein synthesis when a nonsense mutation impairs gene products [9].

The inclusion of fluorine atoms into heterocyclic drug structures represents a tool in medicinal chemistry to boost the activity of bioactive molecules; in fact, the combination of these two features is constantly appearing in new molecular entities with various biological activities, as demonstrated by the increasing number of newly synthesized fluorinated heterocyclic compounds among the Food and Drug Administration’s FDA-approved drugs [10].

About 20% of the anticancer and antibiotic drugs contain fluorine atoms, in particular, five-membered heterocycles and their benzo-fused systems bearing directly connected fluorine atom(s) have shown in vivo and in vitro anticancer and antimicrobial activities, displaying a promising safety index and reduced cytotoxicity in non-cancerous cell lines [11].

In conclusion, collaborative efforts between medicinal chemists, pharmacologists, and computational scientists have accelerated the pace of drug discovery in the heterocyclic space. By leveraging cutting-edge technologies and interdisciplinary approaches, researchers are pushing the boundaries of drug design and uncovering novel therapeutic targets, highlighting that the potential for heterocycles to address unmet medical needs and combat complex diseases is immense.
